# Transcriptomic insights into UTUC: role of inflammatory fibrosis and potential for personalized treatment

**DOI:** 10.1186/s12967-023-04815-y

**Published:** 2024-01-05

**Authors:** Keqiang Li, Zhenlin Huang, Guoqing Xie, Budeng Huang, Liang Song, Yu Zhang, Jinjian Yang

**Affiliations:** 1 Department of Urology, the First Affiliated Hospital of Zhengzhou University, Zhengzhou, Henan Province China; 2 Academy of Medical Sciences, Zhengzhou University, Zhengzhou, Henan Province China

**Keywords:** UTUC, Bladder Cancer, Aristolochic acid, Fibrosis, TKIs

## Abstract

**Background:**

Upper tract urothelial carcinoma (UTUC) is a rare disease, belonging to the same category of urothelial cancers as bladder cancer (BC). Despite sharing similar non-surgical treatment modalities, UTUC demonstrates a higher metastasis propensity than BC. Furthermore, although both cancers exhibit similar molecular disease emergence mechanisms, sequencing data reveals some differences. Our study investigates the transcriptomic distinctions between UTUC and BC, explores the causes behind UTUC's heightened metastatic tendency, constructs a model for UTUC metastasis and prognosis, and propose personalized treatment strategies for UTUC.

**Methods:**

In our research, we utilized differential gene expression analysis, interaction networks, and Cox regression to explore the enhanced metastatic propensity of UTUC. We formulated and validated a prognostic risk model using diverse techniques, including cell co-culture, reverse transcription quantitative polymerase chain reaction (rt-qPCR), western blotting, and transwell experiments. Our methodological approach also involved survival analysis, risk model construction, and drug screening leveraging the databases of CTRPv2, PRISM and CMap. We used the Masson staining technique for histological assessments. All statistical evaluations were conducted using R software and GraphPad Prism 9, reinforcing the rigorous and comprehensive nature of our research approach.

**Results:**

Screening through inflammatory fibrosis revealed a reduction of extracellular matrix and cell adhesion molecules regulated by proteoglycans in UTUC compared with BC, making UTUC more metastasis-prone. We demonstrated that SDC1, LUM, VEGFA, WNT7B, and TIMP3, are critical in promoting UTUC metastasis. A risk model based on these five molecules can effectively predict the risk of UTUC metastasis and disease-free survival time. Given UTUC's unique molecular mechanisms distinct from BC, we discovered that UTUC patients could better mitigate the issue of poor prognosis associated with UTUC's easy metastasis through tyrosine kinase inhibitors (TKIs) alongside the conventional gemcitabine and cisplatin chemotherapy regimen.

**Conclusions:**

The poor prognosis of UTUC because of its high metastatic propensity is intimately tied to inflammatory fibrosis induced by the accumulation of reactive oxygen species. The biological model constructed using the five molecules SDC1, LUM, VEGFA, WNT7B, and TIMP3 can effectively predict patient prognosis. UTUC patients require specialized treatments in addition to conventional regimens, with TKIs exhibiting significant potential.

**Supplementary Information:**

The online version contains supplementary material available at 10.1186/s12967-023-04815-y.

## Introduction

Upper Tract Urothelial Carcinoma (UTUC) is relatively rare worldwide but shows a pronounced regional concentration, particularly in East Asia and the Balkan Peninsula. This phenomenon is primarily associated with regional herbal medicine practices and specific dietary habits, particularly the consumption of aristolochic acid (AA) [1–3]. This phenomenon is intrinsically linked to the inflammatory responses induced by AA-triggered DNA mismatches, and this issue has been accorded substantial attention in various urological guidelines. Although both UTUC and bladder cancer (BC) are transitional cell carcinomas sharing similar clinical therapies [3, 4], their prognoses significantly diverge, with UTUC demonstrating a stronger tendency for metastasis [5]. This subclass of cancer has not received sufficient attention, which leaves the prognostic differences unexplained and prevents further progress in enhancing the UTUC prognosis.

As epidemiology and precision medicine advance, the disparities between these two types of urothelial cancers are being increasingly recognized. Therefore, investigating the differences between UTUC and BC and uncovering the biological mechanisms behind UTUC's poor prognosis holds tremendous clinical implications for improving the prognosis of UTUC patients.

Recently, with the advancement of genomics and transcriptomics technologies, research has increasingly focused on the gene expression profiles and transcriptomic differences of UTUC [6–8]. However, these studies have primarily focused on UTUC's onset mechanism and have not delineated the reasons for the prognostic differences between UTUC and the "classic" urothelial cancers, nor have they proposed specific treatment plans. Thus, based on these studies, we systematically analyzed of the transcriptional differences between UTUC and BC to identify and verify UTUC's unique differentially expressed genes, explore their roles in UTUC pathogenesis, and elucidate the reasons for UTUC's poorer prognosis.

Conversely, extensive research has shown that tumour occurrence, development, and metastasis not only relate to the tumour cells' gene mutations and expression changes but also to modifications in the tumour microenvironment [9, 10]. This microenvironment includes the extracellular matrix around tumour cells, immune cells, vascular cells. These elements interact with tumour cells through complex signalling pathways, influencing the tumour's growth, invasion, and metastatic capacity. Especially for UTUC, the intake of AA can induce renal fibrosis and is likely to stimulate specific changes in the UTUC microenvironment, such as persistent inflammatory response and remodelling of the extracellular matrix. These changes may play an instrumental role in the propensity of UTUC for metastasis and poor prognosis [6, 11].

At present, we find a discernible scarcity of transcriptomic research specifically devoted to UTUC. A sizeable fraction of the existing limited studies does not differentiate or contrast UTUC and BC, both of which are urothelial carcinoma [12, 13]. The minority of studies that have recognised this disparity have not adequately elucidated the reasons for UTUC's comparatively poorer prognosis through focused transcriptomic research [6, 7, 14, 15]. Moreover, these studies have not established links with inflammatory fibrosis, which can be instigated by factors such as aristolochic acid. Accordingly, the present study analysis UTUC's transcriptome, compares the transcriptomic differences between UTUC and BC, identifies UTUC's specific differentially expressed genes, investigates their influence on the biological behaviour of UTUC, and explore how the tumour microenvironment affects UTUC, promoting UTUC's metastasis and progression. Our results can provide a theoretical foundation for developing new treatment strategies for UTUC and improve the prognosis of UTUC patients.

## Results

### Similarities between UTUC and BC, with UTUC showing a higher tendency for metastasis

**Fig. 1:**
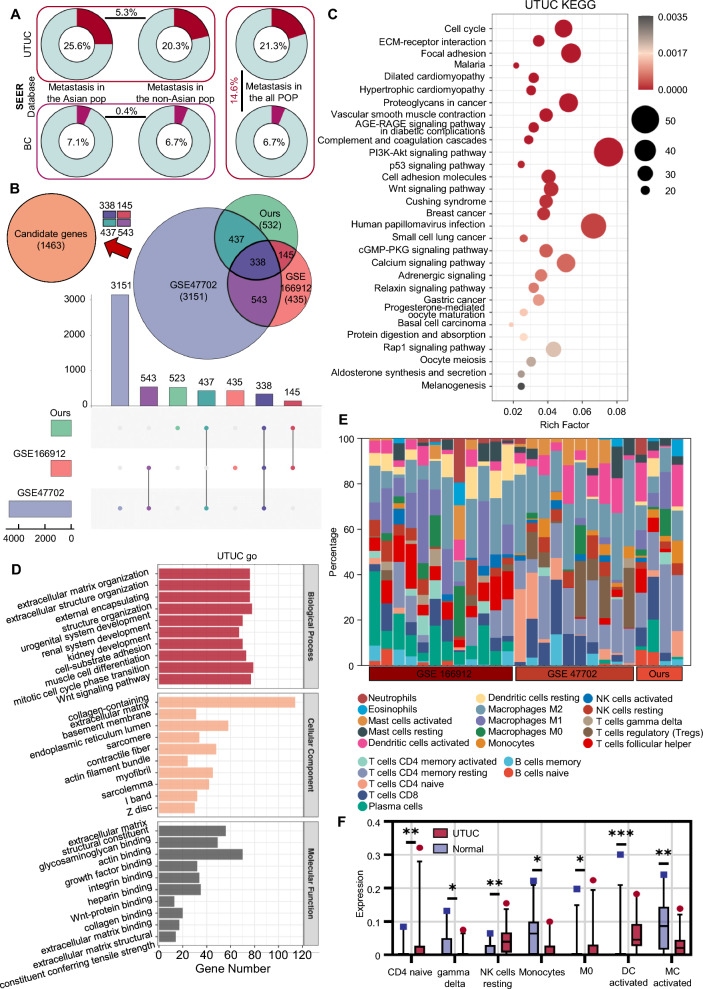
Increased metastasis tendency in UTUC within Asian populations and differential gene enrichment analysis in UTUC. **A** Metastasis rates of UTUC and BC in different populations according to the SEER database. **B** Identification of differential genes between UTUC and adjacent normal tissue. Genes that appear two or more times within the differentially expressed genes across three datasets are selected as candidate genes. **C** KEGG pathway enrichment analysis of differential genes. **D** Gene Ontology (GO) enrichment analysis of differential genes. **E**–**F** Immune infiltration analysis of differential genes in UTUC. (*p* > 0.05 as ns, *p* ≤ 0.05 as *, *p* ≤ 0.01 as **, *p* ≤ 0.001 as ***)

Our clinical experience and data from the SEER database indicate that UTUC is more prone to metastasis with a poorer prognosis (Fig. 1A) (Additional file 2: Table S1) [16]. However, the underlying reasons have remained elusive. Recent studies have ascribed the characteristic of UTUC's easy metastasis to its anatomical differences with BC [17, 18]. Nevertheless, through the SEER database (UTUC: 15,089 cases, BC: 202,868 cases), we found that not only was the incidence of UTUC higher in the Asian region, but also the rate of metastasis was also higher, with an increase of 5% than other regions and a rise of 3.85% than the overall rate (including the Asian region), which significantly diverges from BC (Fig. 1A). The excessive intake of AA because of regional herbal medicine and dietary habits can explain the regional concentration of UTUC incidence, but this reason cannot justify why the rate of metastasis is also significantly increased in these regions. As there are multiple causes of UTUC, such as smoking and Lynch syndrome, we speculate that the specific pathogenic factors of the Asian population not only lead to a higher incidence of UTUC but also cause other changes in UTUC, enhancing its metastasis.

However, as a subclass tumour, the transcriptome sequencing data for UTUC remains limited. To address this issue, we independently conducted transcriptome sequencing on four pairs of UTUC tumours and their corresponding adjacent cancer tissues with a clear history of AA use. We also found only two sets of UTUC transcriptome sequencing data from the Gene Expression Omnibus (GEO) database: GSE19912 (12 tumour samples and 12 normal samples, having a clear history of AA exposure) and GSE47702 (10 tumour samples and 10 normal samples, from regions with high usage of AA-containing herbal medicines).

Based on the provided form of transcriptome data, we used the Dseq2 and limma software packages for differential analysis (LogFC > 1, *p* < 0.05) and generated the corresponding heat maps and volcano plots (Additional file 1: Figure S1A–F). To compare the transcriptomic differences between UTUC and BC, we also performed a differential analysis on the BC transcriptome data from The Cancer Genome Atlas (TCGA) (402 tumour samples and 19 normal samples) (LogFC > 1, *p* < 0.05) and produced the corresponding heat maps and volcano plots (Additional file 1: Figure S2A, B).

Considering the small number of UTUC samples, to increase the credibility of differential genes, we selected differential genes appearing at least twice in three gene sets (our data, GSE19912, GSE47702), and marked them as definite differential gene sets. This set contains 1463 differential genes with higher credibility (Fig. 1B).

We first conducted gene ontology (GO) and Kyoto Encyclopedia of Genes and Genomes (KEGG) enrichment analysis on UTUC differential genes and BC differential genes (Fig. 1C, D and Additional file 1: Figure S3A, B) to reveal the potential functions of these genes and summarize the overall changes in UTUC and BC. We further compared the enrichment results of UTUC and BC. In the KEGG and GO enrichment analyses, UTUC and BC demonstrated significant similarities. As UTUC and BC are both transitional urothelial carcinomas, and their biological behaviors are similar. This also explains why the treatment for UTUC and BC are so alike.

However, in the immune infiltration analysis, the results of UTUC and BC demonstratedsignificant differences (Fig. 1E, F and Additional file 1: Figure S3C, D). We believe this is due to the anatomical location differences and different pathogenic factors between UTUC and BC. UTUC is closely related to the intake of AA and often accompanies the AA-induced inflammatory response. This inflammatory response makes the tumour microenvironment of UTUC significantly different from BC. Numerous studies have confirmed that tumour microenvironment changes significantly affect immune cell infiltration [19–22]. By integrating the transcript data from the three groups, in the immune infiltration detection of UTUC, we observed an increase in the number of naive CD4 + T cells and resting NK cells, a decrease in the number of Gamma delta T cells and activated mast cells, and a decrease in the number of monocytes, but an increase in the number of M0 macrophages and activated dendritic cells.

### Enrichment analysis highlights key differences in proteoglycans, cell adhesion molecules, and the extracellular matrix molecules between UTUC and BC

**Fig. 2:**
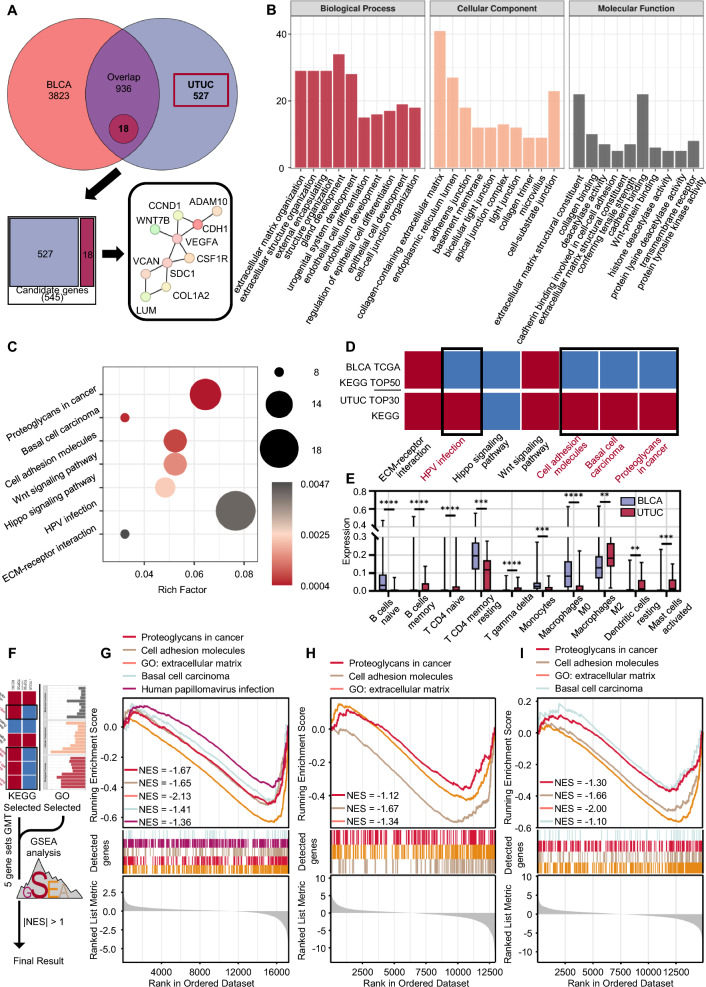
Differential analysis between UTUC and BC. **A** Identification of differential genes between UTUC and BC. **B** Gene Ontology (GO) analysis of UTUC-specific differential genes. **C** KEGG pathway analysis of UTUC-specific differential genes. **D** Screening of UTUC-specific KEGG pathways (those not ranked in the top in BLCA differential gene KEGG but ranked in the top in UTUC differential gene KEGG). **E** Differential immune infiltration analysis between UTUC and BC. **F**-**I** Further GSEA screening of UTUC-specific pathways: Proteoglycans pathway, cell adhesion molecules pathway, and extracellular matrix molecules pathway show significant differences in three groups of UTUC transcriptome data (|NES|> 1). (*p* ≤ 0.01 as **, *p* ≤ 0.001 as ***, *p* ≤ 0.0001 as ****)

Although both UTUC and BC are urothelial carcinomas, and their treatment methods are similar, or identical in some cases, their prognoses significantly differ. To investigate the reasons for this discrepancy and improve the prognosis of UTUC patients, we compared the transcriptomic differences between UTUC and BC. We compared the 4759 differential genes in BC in TCGA and the previously obtained 1463 definite differential genes of UTUC, revealing527 differential genes between UTUC and BC, and 18 differential genes with opposite expression in UTUC and BC (for instance, GeneA in UTUC LogFC > 1; GeneA in BC LogFC < -1). In total, we found 545 differentially expressed genes unique to UTUC (Fig. 2A).

To clarify which changes in UTUC may lead to malignant changes, we built a protein–protein interaction network using the STRING database to identify the hub genes in these 545 differential genes, presenting the top 10 among them (Fig. 2A, Additional file 1: Figure S4A). Then, we performed GO and KEGG enrichment analysis on these 545 genes to study the potential functions of these genes (Fig. 2B, C). Go analysis revealed that these differential genes unique to UTUC were widely enriched in ECM (extracellular matrix) related pathways in GO analysis, while KEGG analysis revealed that “Proteoglycans in cancer”, “Basal cell carcinoma”, “Cell adhesion molecules”, “Wnt signaling pathway”, “Hippo signaling pathway", “HPV infection” and “ECM-receptor interaction" are significantly enriched.

Among them, “Proteoglycans in cancer”, “Basal cell carcinoma”, “Cell adhesion molecules” and “HPV infection” are top-ranked in the KEGG enrichment analysis of UTUC and adjacent tissues, but not significant in the KEGG enrichment analysis of BC and adjacent tissues. We believe that these four screened KEGG pathways and the highly enriched ECM related pathways in GO analysis may be the reasons for the specific biological behavior of UTUC.

To further increase the credibility and confirm the general trend of the decrease of these five pathway gene sets in the UTUC, we performed GSEA analysis (Fig. 2F). The results demonstratedthat proteoglycans, cell adhesion molecules, and ECM are significantly down-regulated in all three transcriptome data sets (|NES|> 1) (Fig. 2G-I), indicating that the tumour microenvironment of UTUC is indeed significantly different from BC. This change may be a key factor in the difference in prognosis between UTUC and BC.

Although AA often causes severe inflammatory fibrosis [23, 24], our analysis results show that the overall degree of fibrosis in UTUC tumours is even lower (Fig. 2G–I, Additional file 1: Figure S3E). Many researches focusing on the relationship between tumours and fibrosis have mentioned that activated fibroblasts can cause remodelling of the extracellular matrix (ECM) [9], thereby affecting the hardness of the primary tumour, directing the induction of tumour cells, enhancing the invasiveness of tumour cells, and tumour cells that break the fibrosis “cage” can obtain a larger survival space and more nutrients, forming a Matthew effect, as the main part of the final solid tumour. In this case, the final solid tumour often has strong metastasis, multifocality, fewer extracellular matrices, and lower fibrosis. This also explains why UTUC is multifocal and more prone to metastasis compared to BC.

This change is also well reflected in the immune infiltration difference between UTUC and BC: compared with BC, the decrease in naive B cells and the rise in memory B cells, the rise in naive CD4 + T cells, the decrease in memory CD4 + T cells and the rise in gamma delta T cells in UTUC indicate a certain persistent inflammatory response. The increase in mast cells may suggest a continuous inflammatory response, whereas the decrease in monocytes and M0 macrophages and the increase in M2 macrophages and activated dendritic cells may be signs of inflammation and tissue repair responses. These finding suggest that a persistent inflammatory stimulus exists in UTUC, which may eventually cause tissue fibrosis and enhance the invasiveness of UTUC. Moreover, due to the tumour-promoting role of M2 macrophages, the increase in M2 macrophages also indicates a poor prognosis (Fig. 2E) [25, 26].

### Significant interactions among proteoglycans, cell adhesion molecules, and extracellular matrix components

Proteoglycans, cell adhesion, and extracellular matrix molecules form an extremely complex and intricate network. These molecules interact with each other, regulating cell behaviour and destiny [27].

**Fig. 3:**
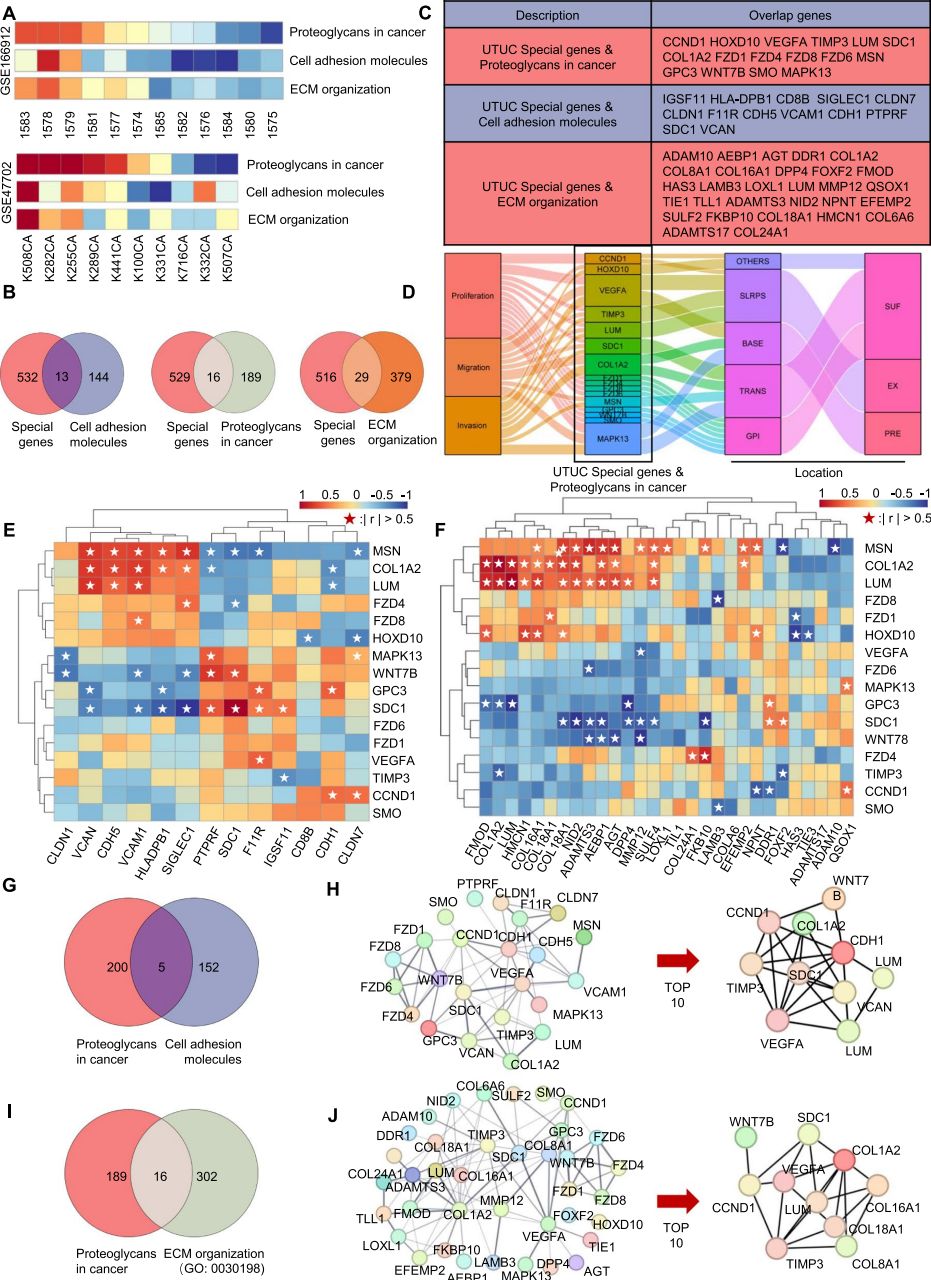
Close association among UTUC-specific differential pathways. **A** ssGSEA analysis shows a positive correlation in two large-sample UTUC datasets (GSE166912, GSE47702): Proteoglycans pathway, cell adhesion molecules pathway, and extracellular matrix molecules pathway. **B**-**C** UTUC-specific differential genes in the three specific pathways. **D** Proteoglycans exhibit diversity, exist in different structures of tumour cells, and participate in tumour proliferation, migration, and invasion. **E**–**F** Co-expression analysis reveals a close association among most UTUC-specific differential genes in the three specific pathways. **G** Few common genes between Proteoglycans pathway and cell adhesion molecules pathway. **H** Association analysis of UTUC-specific differential genes in the Proteoglycans pathway and cell adhesion molecules pathway using the String database, and top 10 associations are analyzed with Cytoscape to identify Hub genes. **I** Few common genes between Proteoglycans pathway and extracellular matrix molecules pathway. **J** Association analysis of UTUC-specific differential genes in the Proteoglycans pathway and extracellular matrix molecules pathway using the String database, and top 10 associations are analyzed with Cytoscape to identify Hub genes

The enriched proteoglycan-related genes include not only protein-coding genes that directly encode proteoglycans (such as SDC1) but also include ligand signalling molecules that interact with receptors (such as VEGFA). These proteoglycan-related molecules participate in the progression of the tumour in different positions and various aspects of the cell. To further clarify the relationship between the three pathways in the UTUC-specific differential genes, considering the sample size, we scored the general expression of proteoglycan pathways, cell adhesion pathways, and ECM pathways in the GSE166912 and GSE47702 gene sets using single-sample gene set enrichment analysis (ssGSEA), and sorted them according to the expression of the proteoglycan pathway gene set in different samples. The three gene sets have a significant positive correlation (Fig. 3A–D), and the correlation analysis of the expression of these three gene sets of molecules in UTUC-specific differential genes in the three gene sets indicated that these genes also have a large number of co-expression relationships (Figs. 3E, F and Additional file 1 Figure S4B, C). Therefore, there is a significant association between these molecules. To further explore the mechanism of action of these molecules, we compared the entire proteoglycan pathway molecules, cell adhesion pathway molecules, and ECM-related pathway molecules in the KEGG and GO databases. Initially, the findings indicate a limited overlap of genes among these three pathways. (Fig. 3G, I). However, through the association analysis of the STRING database, whether a physical association or an indirect association, the connection between the proteoglycan pathway and the other two is very tight (Additional file 1: Figure S4D, E). This finding shows that many connections between molecules are formed by the direct or indirect influence. Therefore, we used the Cytoscape to further analyze the relationship between the proteoglycan pathway molecules in the UTUC-specific differential genes and the cell adhesion pathway and ECM pathway molecules, and selected the top 10 hub genes for future study (Fig. 3H, J).

### Core differential molecules SDC1, LUM, VEGFA, TIMP3, and WNT7B are closely associated with metastasis and prognosis in UTUC patients

To identify the key genes that play a critical role in the poor prognosis of UTUC, we comprehensively considered the hub genes in the UTUC-specific differential genes (Fig. 2A); the top 10 hub genes in the proteoglycan and cell adhesion pathways, as well as the ECM pathway (Fig. 3H, J); the co-expression characteristics of the three pathways in UTUC-specific differential genes in the GSE166912 and GSE47702 gene sets. We found that some genes repeatedly appear in different comparisons. After comprehensive evaluation, we selected SDC1, LUM, VEGFA, TIMP3, CCND1, WNT7B, and COL1A2 as candidate genes.

**Fig. 4:**
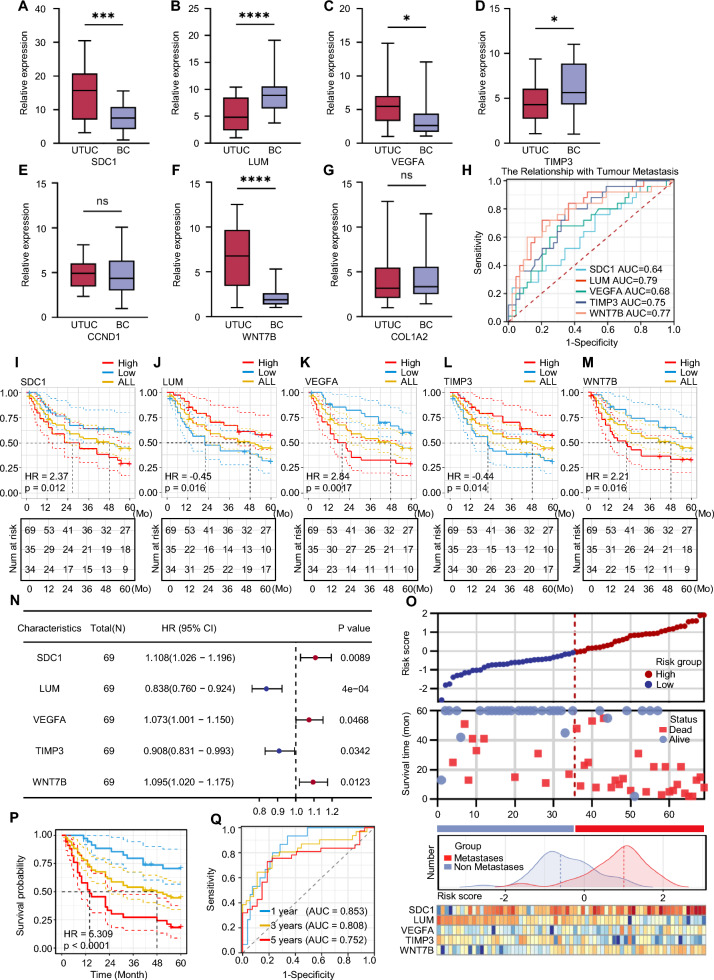
Core specific genes and their impact on prognosis. **A**-**G** Differences in SDC1, LUM, VEGFA, TIMP3, CCND1, WNT7B, COL1A2 expression levels between UTUC and BC. **H** ROC analysis of the association of expression levels of SDC1, LUM, VEGFA, TIMP3, WNT7B with the metastatic propensity of UTUC. **I**-**M** Relationship between the expression levels of SDC1, LUM, VEGFA, TIMP3, WNT7B and the prognosis of UTUC. **N**–**O** Establishment of a risk scoring model via COX regression analysis and categorization of patients into high-risk (red) and low-risk (blue) groups; observation of expression patterns of five signature genes and overall metastasis in both groups. **P** Association between high-risk and low-risk groups with UTUC prognosis. **Q** ROC analysis of the predictive ability of risk scores for 1-year, 3-year, and 5-year prognosis in UTUC patients. (*p* > 0.05 as ns, *p* ≤ 0.05 as *, *p* ≤ 0.001 as ***, *p* ≤ 0.0001 as ****)

We validated these genes in 69 UTUC samples. First, we randomly selected 25 UTUC samples and 25 BC samples and tested the expression levels of these seven candidate genes. The results demonstrate that the expression differences of SDC1, LUM, VEGFA, TIMP3, and WNT7B are significant (*p* < 0.05) (Fig. 4A–G).

Our pan-cancer analysis of these five genes also showed that SDC1, VEGFA, and WNT7B have higher expression in most cancer tissues than in adjacent tissues, whereas LUM and TIMP3 have lower expression in most cancer tissues than in adjacent tissues (Additional file 1: Figure S5A–E).

To further verify whether these genes are related to cancer metastasis and may affect patient survival, we tested the expression levels of these five genes in the remaining 44 UTUC tumour samples. Combined with the expression levels of the previous 25 samples, we divided the samples into two groups based on the high and low expression levels of the genes and analysed the effect of these five genes on survival outcomes. Simultaneously, we used ROC curves to analyse the relationship between gene expression and tumour metastasis.

The survival analysis and ROC analysis results show that all five genes are closely related to the patient's survival (Fig. 4I–M) and tumour metastasis (Fig. 4Q). To increase the practicality of the research results, we used univariate Cox regression analysis of these five genes. The results showed that these five genes were not only related to survival but also significantly different and devoid of collinearity (Additional file 3: Table S2). Considering the few covariates, to avoid overfitting of multivariate analysis, we directly constructed a risk model with existing results, and assigned a risk score for each tissue sample.

(Riskscore = ExpSDC1 × 0.06290643–ExpLUM × 0.1229299 + ExpVEGFA × 0.0428134—ExpTIMP3 × 0.0721954 + ExpWNT7B × 0.0728519),

We divided the samples into high-risk and low-risk groups according to the risk score. The survival curve shows that compared with the low-risk group, the survival rate of the high-risk group is significantly lower, and the metastasis rate is significantly higher (Fig. 4N–P).

Finally, the results of the time-dependent ROC curve show that the risk model had good effects on predicting survival at 1 year (AUC = 0.853), 3 years (AUC = 0.808), and 5 years (AUC = 0.752) (Fig. 4Q).

### Tumour cells escaping from inflammatory fibrosis exhibit significant alterations in the five core differential molecules and enhanced invasion and migration capabilities

We also carried out cell experiments to validate our novel idea that the inflammatory fibrosis process accompanying the early tumour formation of UTUC has a screening effect on tumour cells, eventually leading to UTUC's poorer prognosis compared to BC. The five molecules (SDC1, LUM, VEGFA, TIMP3 and WNT7B) as biomarkers can well predict the poor prognosis of UTUC patients, and these five molecules are likely to participate directly in the process of poor prognosis of UTUC.

**Fig. 5:**
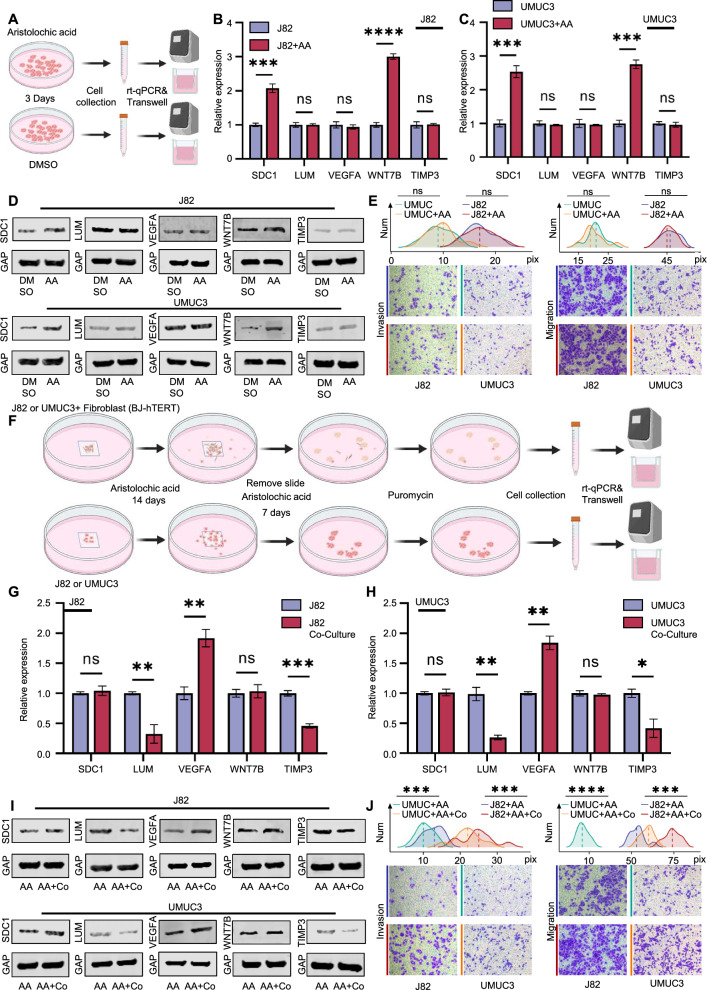
Impact of fibrosis on the migration and invasion abilities of tumour cells. **A**-**D** Following Aristolochic acid stimulation, both J82 and UMUC3 cell lines show a significant increase in WNT7B and SDC1 expression, no significant changes in LUM, VEGFA, TIMP3 expressions. **E** No significant changes in migration and invasion abilities in J82 and UMUC3 after Aristolochic acid stimulation. **F**–**I** After Aristolochic acid stimulation and co-culturing with fibroblasts followed by puromycin selection: significant decrease in LUM, TIMP3 expressions and a significant increase in VEGFA expression in both co-cultured cell lines, with no significant changes in WNT7B and SDC1 levels. **J** Significant changes in migration and invasion abilities in J82 and UMUC3 after Aristolochic acid stimulation and co-culturing with fibroblasts followed by puromycin selection. (*p* > 0.05 as ns, *p* ≤ 0.05 as *, *p* ≤ 0.01 as **, *p* ≤ 0.001 as ***, *p* ≤ 0.0001 as ****)

Based on the expression of SDC1, LUM, VEGFA, TIMP3 and WNT7B in various urothelial carcinoma cell lines in the CCL database, we selected J82 and UMUC3 as the verification cell lines (Additional file 1: Figure S6A), and marked them with puromycin resistance labelling. In these two cell lines, compared with the DMSO group, the expression of WNT7B and SDC1 in the AA stimulation group was significantly different (*p* < 0.01), and the differences in expression in LUM, VEGFA, and TIMP3 were not significant (Fig. 5A–D). Notably, the migration and invasion abilities of the two cell groups after AA treatment showed no difference (Fig. 5E). To further verify the influence of fibroblasts, we co-cultured J82 and UMUC3 with fibroblasts in culture medium containing AA to simulate the process of inflammatory fibrosis. We collected the cells migrated from the co-culture group and the control group. The results showed that the expression of VEGFA increased in the co-culture group (*p* < 0.01), whereas LUM and TIMP3 decreased (*p* < 0.05). The results also indicated that there were no significant differences in the expression of SDC1 and WNT7B between the two groups (Fig. 5F–I). Furthermore, the migration and invasion ability of the co-culture tumour cells was significantly higher than that in the control group (Fig. 5J).

### Tyrosine kinase inhibitors show great potential for precision medicine in UTUC

**Fig. 6:**
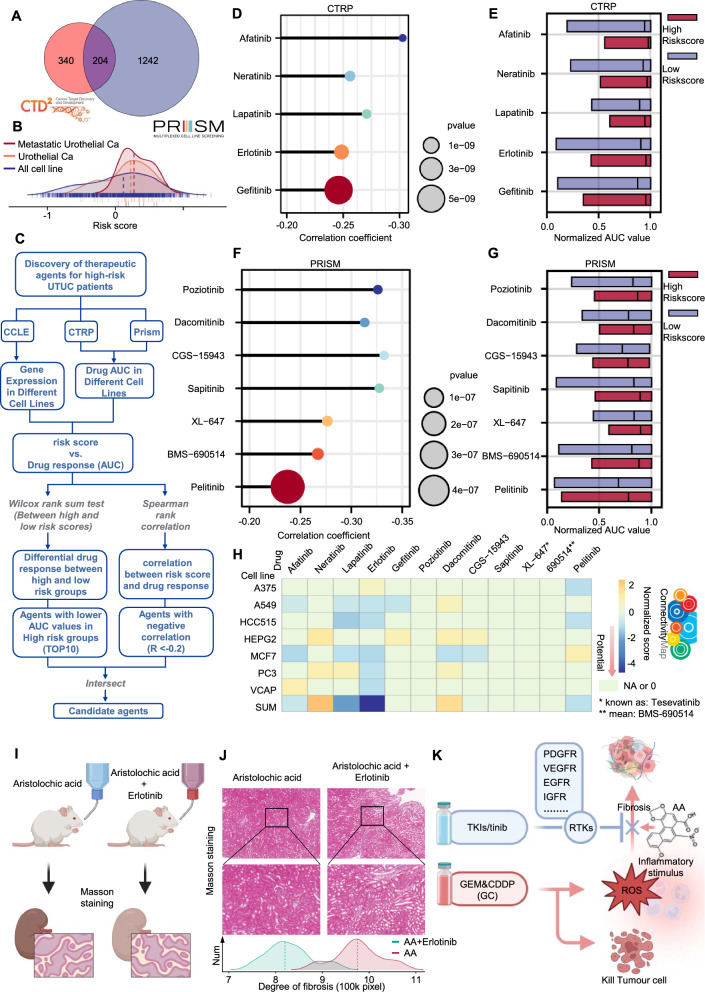
Tyrosine kinase inhibitors demonstrate significant potential in UTUC treatment. **A** Both CTRPv2 and Prism share 204 common drugs. **B** Risk scores for all cell lines in the CCL database, urothelial cell lines, and metastatic urothelial cell lines. **C** Drug screening methodology for CTRPv2 and Prism. **D** Results of shared expression analysis (r < -0.2, Spearman correlation) between selected drugs from CTRPv2 and risk scores. **E** Normalized AUC values for drugs selected by CTRPv2 in high-risk and low-risk groups. **F** Results of shared expression analysis (Spearman correlation, r < -0.2) between selected drugs from Prism and risk scores. **G** Normalized AUC values for drugs selected by Prism in high-risk and low-risk groups. **H** Validation of drug selection results from CTRPv2 and Prism in the CMap database, to identify more potential drugs for targeted UTUC treatment. **I**, **J** Identification of kidney fibrosis in the Aristolochic acid induced kidney disease mouse model using Masson's trichrome staining in the Erlotinib and saline groups. **K** Potential mode of action for tyrosine kinase inhibitors. (*p* ≤ 0.05 as *, *p* ≤ 0.01 as **, *p* ≤ 0.001 as ***, *p* ≤ 0.0001 as ****)

We used the drug sensitivity data from the CTRPv2 and PRISM and the transcriptome expression spectra of the CCL cell lines included in the two databases to construct a drug response prediction model. Considering that UTUC is a solid malignancy, to increase the specificity of the results, we first excluded non-tumour, blood lymphoma, and unspecified cell line transcript expressions from the CCL cell expression spectra. After excluding these cell line expression spectra, the CTRPv2 drug sensitivity dataset contained drug response spectra for 544 compounds and 690 cell line expressions; PRISM contained 1446 compounds and 482 cell line expressions. The CTRPv2 and PRISM datasets contained 204 compounds common to both databases. As the number of cell lines in the CTRPv2 drug sensitivity dataset is more than PRISM, we chose to further analyse these 204 compounds using the CTRPv2 data (Fig. 6A). Meanwhile, after excluding compounds with NAs > 25% in both datasets, the CTRPv2 dataset contained 394 compounds, and 861 compounds in the PRISM dataset contained 861 compounds.

To identify specific drugs for UTUC's characteristics, we utilized the risk model, comprising the five biomarkers, to perform risk scoring on the standardized cell transcriptome data in CEL. Through the score, we found that the comprehensive score of urothelial carcinoma was much higher than the average, and the score of metastatic urothelial carcinoma was higher than that of non-metastatic urothelial carcinoma (Fig. 6B).

We divided all scores into high and low groups, calculated the difference in drug AUC values between the high and low groups, and used the Wilcox test to determine its significance (*p* < 0.05). We then selected the top 10 compounds with negative LogFC values in the two groups, performed the Sperman correlation analysis on the AUC values of these compounds and the risk score, and chose compounds with negative correlation coefficients (r < − 0.20).

In CTRPv2, we finally screened out five drugs: Afatinib, Neratinib, Lapatinib, Erlotinib, Gefitinib, while in PRISM, we finally screened out seven drugs: Poziotinib, Dacomitinib, CGS-15943, Sapitinib, XL-647, BMS-690514, Pelitinib (Fig. 6C–G). Despite these compounds demonstrate increased drug sensitivity under high-risk conditions, this evidence in isolation does not conclusively substantiate that they can provide a supplementary intervention for the unique characteristics of UTUC, particularly its predisposition for swift metastasis and its typically poor prognosis.

Therefore, we used other methods for predictive analysis to confirm the credibility of the screened drugs. We used CMap to establish a UTUC-specific gene expression pattern, analysed and found those compounds that are opposite to the UTUC-specific gene expression pattern (that is, those compounds that have a tendency to return to normal after drug treatment of UTUC-specific differential genes). Among these compounds, Erlotinib, Lapatinib, Pelitinib have comprehensive standardized CMap scores < − 1, which means that these three compounds have greater therapeutic potential (Fig. 6H).

Notably, most of these drugs are tyrosine kinase inhibitors (TKIs). Considering that the specific biological behavior of UTUC is closely related to the inflammatory fibrosis caused by reactive oxygen species (ROS) accumulation, to understand the significance of TKIs in the analysis, we conducted a comprehensive literature search on PubMed to find the characteristics of this kind of drug. The results show that TKIs have been widely proven to have anti-fibrotic effects, which has been verified in fibrosis of the lungs, liver, and kidneys, etc. [28–30]. This finding may also explain why TKIs repeatedly appear in our analysis. We further verified Erlotinib, the drug with the highest score in the analysis results, in a mouse model of AA nephropathy. The Masson staining showed that the degree of renal fibrosis in the Erlotinib group mice was lower than that in the saline group (Fig. 6I, J).

These findings highlight the great potential of TKIs, especially Erlotinib, in the specific treatment of UTUC. In addition to conventional cisplatin and gemcitabine treatment regimens, the combined use of TKIs may effectively inhibit the inflammatory fibrosis caused by normal tissue damage, thereby improving the prognosis of UTUC patients (Fig. 6K).

## Discussion

### The difference between upper tract urothelial carcinoma and bladder cancer

UTUC and BC both belong to transitional cell carcinoma, and in many cases, these two types of cancer are considered as two subtypes of transitional cell carcinoma occurring in different locations. Correspondingly, the clinical treatment methods for these two are identical.

With the wide development of epidemiological research since the twenty-first century, people's understanding of diseases has also improved. Researchers have found that although UTUC and BC have the same origin, UTUC is more prone to metastasis, and its survival outcome is worse. This finding is supported by database data and our actual clinical experience: there are many bladder cancer patients who have been receiving non-surgical treatment for a long time, and their tumours do not progress; however, similar cases of non-progression under non-surgical treatment in UTUC patients are very rare.

In addition, epidemiological research have shown that the causes of the two tumour are different, especially UTUC is closely related to the exposure to AA, accordingly, UTUC patients have a strong aggregation. In the Asian region where AA-containing herbal medicines are widely used and the Balkan Peninsula where AA-containing foods are consumed, the probability of UTUC occurrence significantly increases. This finding has already received attention from various guidelines. Interestingly, our research on the metastasis of different ethnic UTUCs in the SEER database found that the Asian population not only had a higher incidence but also a higher metastasis rate. The Asian population always had the habit of using AA-containing herbal medicines, so this special pathogenic factor not only increases the incidence of UTUC but also may significantly increase its metastasis rate, thereby worsening prognosis.

### Inflammatory fibrosis leads to poor prognosis of UTUC

Through the analysis of the transcriptomic differences between UTUC and BC, we found that their differences mainly focus on proteoglycans, cell adhesion factors, and the extracellular matrix, which are all essential components of the tumour microenvironment. Moreover, numerous studies on the prognosis of UTUC have highlighted the role of AA in inducing DNA mismatch, which ultimately leads to cellular transformation. This process also results in the accumulation of ROS, triggering severe inflammatory responses. This process is widely recognized as a significant factor in the AA-induced chronic inflammatory kidney disease and renal fibrosis. Notably, fibrosis often precipitates changes in the tumour microenvironment. Consequently, it is logical to associate these alterations in the tumour microenvironment with the AA-induced inflammatory fibrosis. In short, the AA-induced inflammatory fibrosis changed the tumour microenvironment of transitional epithelial carcinoma cells, making two identical origin tumours behave differently in biology.

Specifically, because of inflammation, a large number of fibroblasts are activated, which plays a screening role for tumour cells. As gene mutations are non-directional, some tumour cells have more specific proteoglycans, fewer cell adhesion factors, and less extracellular matrix content, so these cells can escape from the “cage” of fibroblasts and obtain more growth space and more nutrients.

Finally, cells that escaped from the "cage" gradually occupied a dominant position in UTUC. Because of the change of proteoglycans and the decrease of extracellular matrix and adhesion factors, these solid tumours have fewer fibrous components and have a higher possibility of metastasis than BC.

Concurrently, immune cells also responded to the persistent inflammation and fibrosis caused by AA, especially the increase of M2 macrophages, which may also promote the poor prognosis of UTUC.

**Fig. 7:**
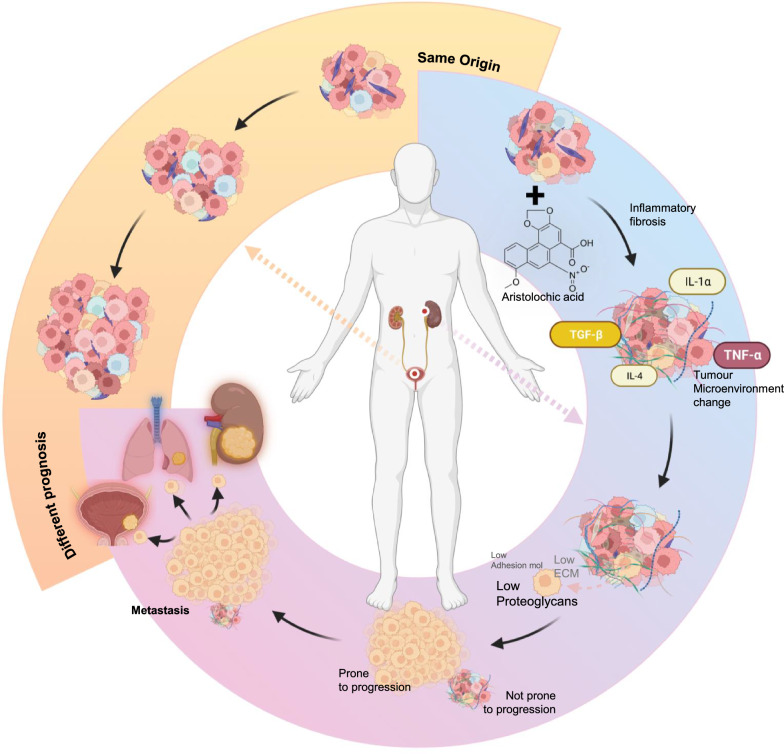
Inflammatory fibrosis results in prognostic differences between UTUC and BC

In conclusion, the inflammatory fibrosis caused by ROS accumulation is a behaviour that inhibits tumour growth during the early tumour stage, but due to the directional screening of non-directional mutations of tumour cells and the change of the immune microenvironment, fibrosis has become an unignorable factor of poor prognosis. This is also why, compared to BC, the higher the T stage of UTUC, the worse the prognosis. Considering the other causes of UTUC, such as smoking, abuse of analgesics, and occupational exposure, etc., all have a close relationship with fibrosis of the upper tract and its surrounding areas, we propose that fibrosis constitutes a significant reason for the starkly different prognoses observed in BC and upper urinary tract urothelial carcinoma, both of which belong to the category of urothelial cancer (Fig. 7).

### Personalized treatment of upper tract urothelial carcinoma

For UTUC and BC, current guidelines suggest the same non-surgical treatment plan, gemcitabine and cisplatin, mainly because they have the same origin and both are urothelial cancers. However, considering their different etiologies and biological behaviours, we propose that implementing specialized treatments, in addition to traditional therapeutic approaches, for patients with UTUC could significantly enhance patient prognosis. Especially considering that many non-surgical treatment plans could cause tissue injuries and trigger ROS accumulation, which might exacerbate the inflammatory response, stimulate the activation of fibroblasts, and increase the chance of metastasis in UTUC cells. Through a comprehensive analysis of multiple databases, we found that TKIs have good potential as a supplement to chemotherapy. It's worth noting that TKIs have been widely proven to have anti-fibrotic effects in many studies in different tissues (including the kidney). This is an important reason why TKIs show promising application prospects for UTUC. On the one hand, TKIs can inhibit the tissue inflammatory fibrosis caused by AA; on the other hand, when used in combination with gemcitabine and cisplatin, TKIs can also alleviate the possibility of upper tract tissue fibrosis caused by gemcitabine and cisplatin chemotherapy. We must point out that the databases we have analysed for this study exclusively contain information related to oncology therapeutics, and thus lacked data on certain routine medications such as antibiotics. We have reasons to believe that as long as a certain drug can suppress inflammation and prevent excessive fibrosis, it may have a certain effect on improving the prognosis of UTUC.

## Conclusion

The poor prognosis of UTUC because of its high metastatic propensity is intimately tied to inflammatory fibrosis induced by the accumulation of reactive oxygen species.

The biological model constructed using the five molecules SDC1, LUM, VEGFA, WNT7B, and TIMP3 can effectively predict patient prognosis.

UTUC patients require specialized treatments in addition to conventional regimens, with TKIs exhibiting significant potential. 

## Method

### Data collection

In our study, the epidemiological data of UTUC and BC came from the Surveillance Research Program, National Cancer Institute SEER*Stat software version v8.4.2. (Incidence SEER research data 18 Registries, Year of diagnosis from 2004 to 2015, Locations: UTUC: C65.9, C66.9; BC: C67.0–C67.9 Pathology reports only from autopsies or death certificates were excluded). RNA-seq data from UTUC and cancer-adjacent tissues were gathered from the GEO database and our independent collection of four UTUC patients with a clear history of aristolochic acid intake. RNA-seq data for BC and cancer-adjacent tissues were obtained from the TCGA database Drug sensitivity data (AUC) came from the CTRPv2 and PRISM databases, and the RNA-seq for different cell lines in the two databases were collected from the CCL database. We used the IDA and the IDACombo methods (Simplicity1) to standardise the AUC values of different drugs in different cell lines [31–34]. The UTUC validation data samples came from the 69 pairs of tissue samples collected by the Department of Urology, the First Affiliated Hospital of Zhengzhou University from 2017 to 2018 (The pathological characteristics of UTUC patients are presented in Additional file 3: Table S2.).

### Differential analysis, functional enrichment analysis, PPI network construction

We used the “limma” or “DEseq2” R package to identify transcriptome differential expression between tumour and adjacent solid tissue samples in GEO and TCGA dataset. We used the “clusterProfiler” R package for gene ontology (GO) enrichment analysis and Kyoto Encyclopedia of Genes and Genomes (KEGG) pathway enrichment analysis. To further clarify the differential pathways between UTUC and BC, we also used the “clusterProfiler” R package for GSEA analysis. At the same time, we used the “GSVA” R package to perform single-sample gene set enrichment analysis (ssGSEA) to preliminarily determine the association between three UTUC-specific related pathways. To further clarify that UTUC-specific genes inthree pathways also have a close co-expression relationship, we used the Spearman and Stats R package to test the association degree of the UTUC-specific genes in the three pathways. To further determine the interaction between these genes, we uploaded them to the STRING database and got their connections, and then used Cytoscape software to build a PPI network and calculate the hub genes within it [35, 36].

### RNA isolation and quantitative PCR (RT-qPCR)

We used TRIzol® reagent (Invitrogen) to extract Total RNA. We prepared cDNA using NovoScript® Plus All-in-one 1st Strand cDNA Synthesis SuperMix (gDNA Purge) (E047, Novoprotein, Shanghai, China). We quantified gene transcription using the QuantStudio Three Real-Time PCR System (Thermo Fisher) and NovoStart^®^ SYBR Two-Step qRT-PCR Kit (E093, Novoprotein, Shanghai, China), with GAPDH as an internal control (The primers used for rt-qPCR, along with their corresponding sequences, are listed in Additional file 3: Table S2).

### Survival analysis and risk model construction

We categorized the UTUC tissue samples into two groups, high expression and low expression, based on the expression levels of the five core differential genes (SDC1, LUM, WNT7B, VEGFA, and TIMP3). We used the “survminer” R package to analyse the high expression and low expression groups of the five genes respectively, and also used univariate Cox analysis to study the prognostic value of these five specific genes. Due to the small number of variables, to prevent overfitting, we directly used the Cox analysis results to construct a risk model. The algorithm for patient construction is as follows:

$RiskScore\, = \,\sum\nolimits_{i = 1}^{n} {\exp i\, \times \,\beta i}$ (expi is the expression of each specific differential gene, and βi is the regression coefficient of the specific differential gene)

By categorizing the samples into low-risk and high-risk groups according to the risk score, we observed the metastasis situation and the expression of five specific differential genes during the change of risk score.

We used the “survival” and “timeROC” R package to calculate the association between five specific differential genes and tumour metastasis using the ROC curve, and we used time-dependent ROC to test the predictive ability of the risk model for UTUC patients’ prognosis in 1, 3, and 5 years.

### Western blotting (WB)

We used the RIPA buffer (containing 1% PMSF) provided by Solarbio, (Beijing, China) to extract total protein from tissues and cells. We used the BCA protein quantitation kit from Solarbio to quantify the protein. We used the PAGE gel rapid preparation kit (from Epizyme, Shanghai, China) to prepare SDS-PAGE gel and separated the total protein (20 μg) in the gel. We then rapidly transferred the protein to the nitrocellulose membrane from Millipore. For better binding with the antibody, we blocked the nitrocellulose membrane with blocking solution at room temperature for 1 h. The nitrocellulose membrane was incubated overnight with the primary antibody, washed with Tris-buffered saline containing Tween, and then incubated with goat anti-rabbit IgG or goat anti-mouse IgG. We used the Odyssey CLx infrared imaging system from Gene Company Limited (Gene Company Limited, Hong Kong, China) to detect the target protein bands. The antibodies used are presented in Additional file 3: Table S2.

### Cell culture and transfection

We obtained J82 and UMUC3 from Procell (Procell Life Science &Technology Co., Ltd., Wuhan, China). BJ cells were obtained from ATCC (ATCC LGC Standards GmbH, located in Manassas, Virginia, USA; catalog number CRL-2522). We prepared BJ-hTERT cells by transduction using the pWZL-Blast-Flag-HA-hTERT retroviral vector (Addgene; catalog number 22396). We cultured them using high-glucose DMEM medium (containing 10% fetal bovine serum, 1% penicillin and streptomycin). To facilitate the screening of fibroblasts and urothelial carcinoma cells, we transfected urothelial carcinoma cells with puromycin resistance gene retroviral vector.

### Cell migration and invasion ability test

Cell migration and invasion ability were also assessed using the 8μm pore size transwell assay (Corning Inc., Corning, NY, USA). After cells were digested with trypsin, they were collected and placed in the upper chamber containing 1 × 10^5^ cells (matrix gel was added for the invasion experiment), and then the medium in each well was supplemented to 200 μL. The upper chamber was placed in a 24-well plate with DMEM medium containing 20% FBS. After incubation at 37 °C and 5% CO_2_ for 24 h, the upper chamber was stained, observed and analysed.

### Cell co-culture

After cells were digested with trypsin, they were placed in a 6-well plate containing a square cell climbing slice (24 mm) at a ratio of 1:10 (2.5 × 10^5^ BJ-hTERT, 2.5 × 10^4^ J82 or UMUC3) [37]. After the cells were attached, four glass slides were taken out and combined and placed in a 15cm culture dish with DMEM containing AA (SJ-MN0173, Shandong Sparkjade Biotechnology Co.,Ltd.), the medium (containing AA) was changed every 3 days, and the cells were taken out after 14 days. Small clones of urothelial carcinoma cells can be observed far away from the cell climbing slice. The cells were continued to culture for 7 days, then puromycin (T19978, TargetMol, USA) was added for screening for 24 h, change the medium (containing AA) and continue to culture for 24 h, then collect cells for next step analysis.

### Drug screening and aristolochic acid nephropathy mouse model drug validation

We used the CTRPv2 and PRISM databases for drug screening, and the screening results were secondarily verified using the Connectivity map (CMap) across its seven core cell lines [38].

C57BL/6 wild-type mice (male, 20g ± 3g) were intraperitoneally injected with aristolochic acid 5 mg/kg/d for 7 days, to complete the construction of the aristolochic acid nephropathy model. The mice were divided into two groups, one group was gavaged with Erlotinib (T0373, TargetMol, USA), 50 mg/kg/d, and the other group was gavaged with saline, for a duration of 2 weeks.

### Masson staining

Wax sections were sequentially placed in eco-friendly dewaxing solutions I and II, processed in anhydrous ethanol I and II, treated in 75% alcohol, and washed with tap water. Frozen sections were removed from the − 20°C refrigerator to recover to room temperature, fixed with tissue fixing solution, and washed with running water. The sections were soaked in Masson A solution overnight and then washed with tap water. Then the sections were soaked in mixed Masson B and Masson C dye solution, then washed with tap water, and then differentiated with differentiation solution, and again washed with tap water. The sections were soaked in Masson D solution for staining, then washed with tap water. Then the sections were soaked in Masson E solution for staining. Without washing, the sections were slightly drained and directly placed in Masson F solution for staining. The sections were differentiated by acetic acid washing, and then dehydrated in two vats of anhydrous ethanol. Finally, the sections were placed in the third vat of anhydrous ethanol for processing, then treated in xylene for clarification, and finally sealed with neutral gum. After completing the above steps, the sections were checked under a microscope and ImageJ software was used for image capture and analysis.

### Statistical analysis

All statistical analyses and graph presentations were conducted using R software (Version 4.0.3–4.2.3) and GraphPad Prism 9. The significance of variables was analyzed using either t-tests or Wilcoxon tests.

### Supplementary Information

**Additional file 1: ** Supplementary Figures.

**Additional file 2:** Pathological information related to tumor metastasis of UTUC and BC in the SEER database.

**Additional file 3:** Antibodies, Primers, and Tissue Sample Information.

## Data Availability

All the data supporting this study's findings are available from the corresponding authors upon reasonable request. The raw fastq data and matrix of bulk RNA-seq will be deposited in the GEO public database repository after acceptance.
