# Supplementary material for: Transcriptomic insights into UTUC: role of inflammatory fibrosis and potential for personalized treatment
Source: J Transl Med. 2024 Jan 5;22:24. doi: 10.1186/s12967-023-04815-y (PMC10768331; doi:10.1186/s12967-023-04815-y)
Supplement: Supplementary file 1 — Additional file 1: Supplementary Figures. [file 12967_2023_4815_MOESM1_ESM.pdf]

Inflammatory Fibrosis: The Culprit behind Poor Prognosis in Upper Tract Urothelial Carcinoma

Keqiang Li , Zhenlin Huang, Guoqing Xie, Budeng Huang, Liang Song, Yu Zhang, Jinjian Yang

Information

Supplementary Figure 1 （Supplemental to Figure. 1）

Differential gene analysis across three datasets

Supplementary Figure 2 （Supplemental to Figure.2）

Differential gene analysis in TCGA-BLCA

Supplementary Figure 3 （Supplemental to Figure. 2）

Enrichment analysis and immune infiltration of BLCA differential genes

Supplementary Figure 4 （Supplemental to Figure. 2, 3）

Supplementary figures for correlation analysis

Supplementary Figure 5 （Supplemental to Figure. 4）

Pan-cancer analysis of UTUC-specific differential genes

Supplementary Figure 6 （Supplemental to Figure. 5）

Screening of urothelial carcinoma cell lines

Supplementary Figure 1

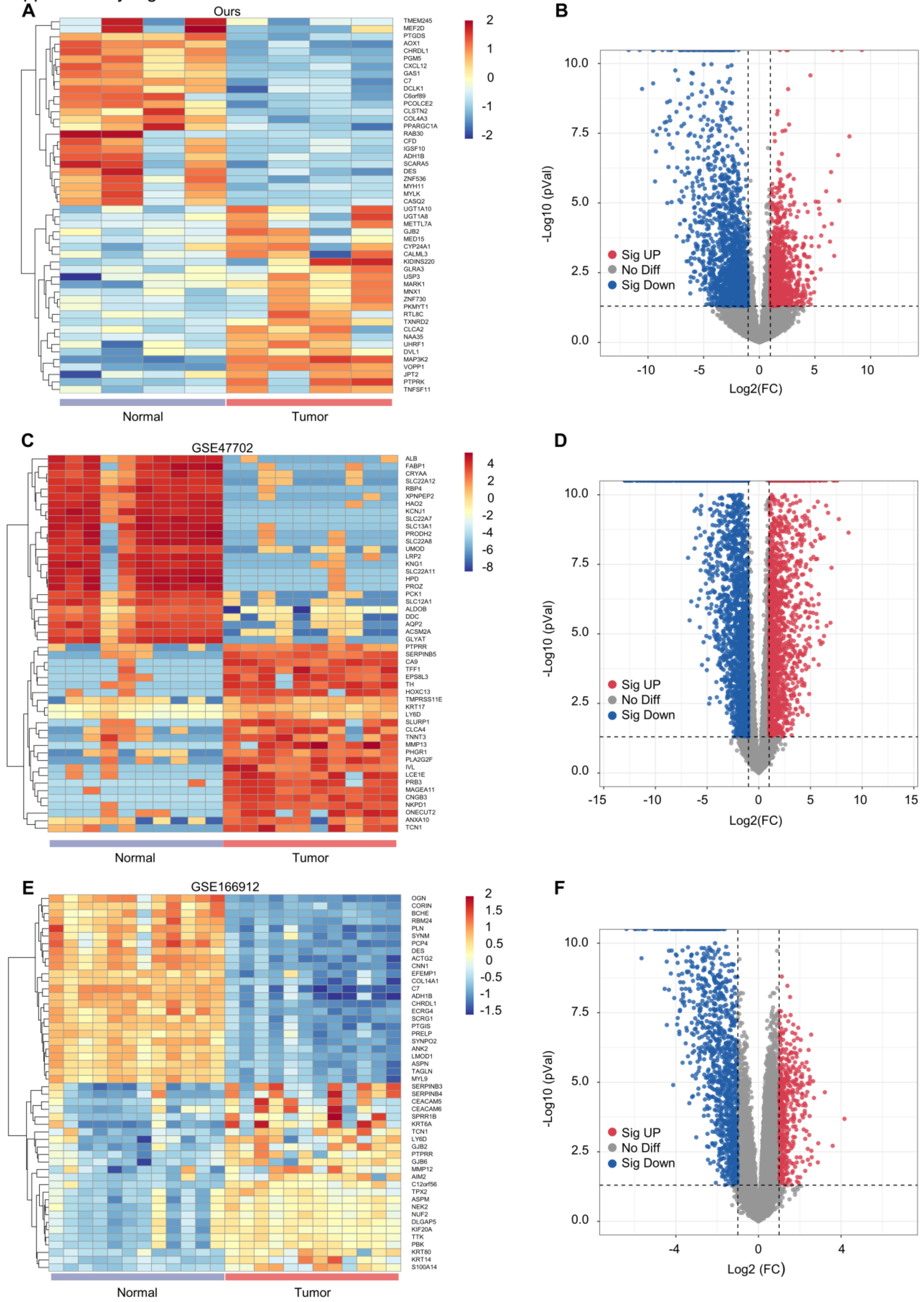

Supplementary Figure 1. Differential gene analysis across three datasets.
(A, B) Heatmap and volcano plot of differential genes in UTUC patient data with a history of Aristolochic acid intake that we collected. (C, D) Heatmap and volcano plot of differential genes in GSE47702. (E, F) Heatmap and volcano plot of differential genes in GSE166912.

Supplementary Figure 2

A

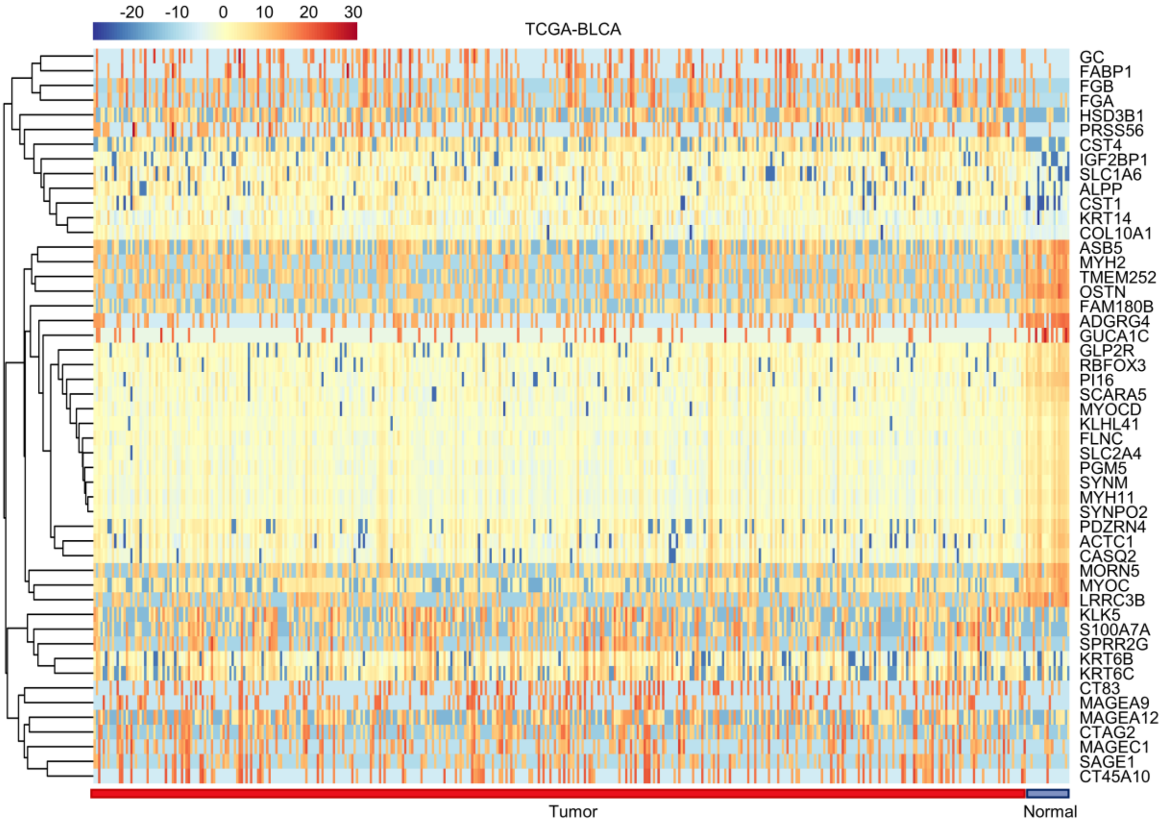

B

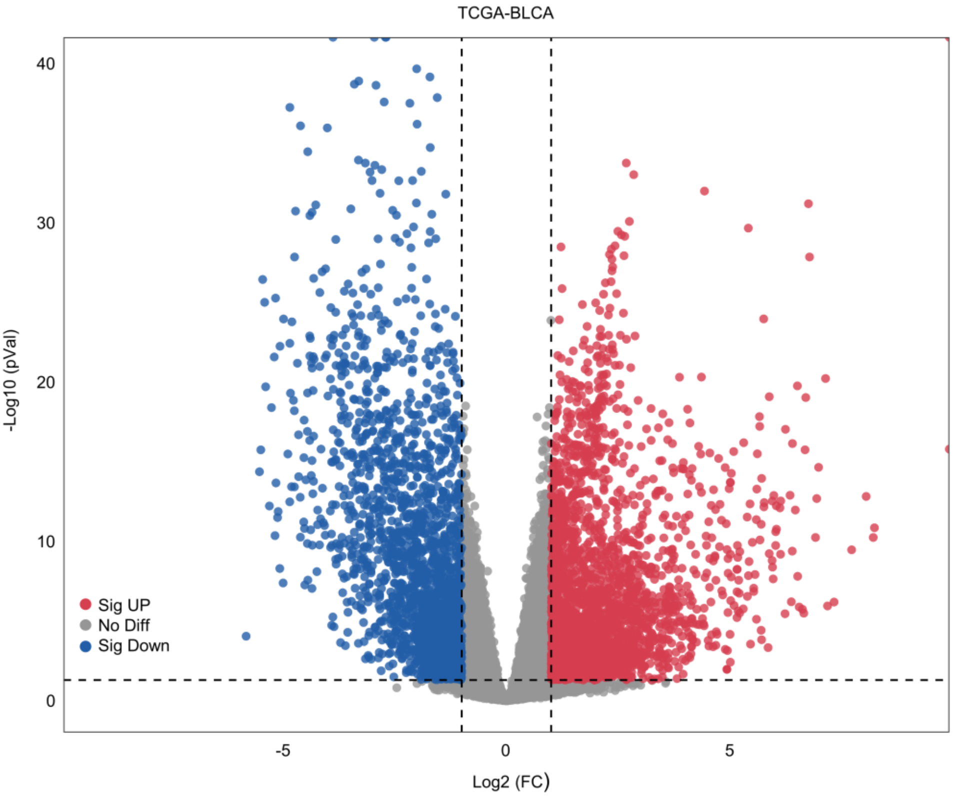

Supplementary Figure 2. Differential gene analysis in TCGA-BLCA.

(A) KEGG enrichment analysis of differential genes in TCGA-BLCA. (B) Go enrichment analysis of differential genes in TCGA-BLCA. (C, D) Immune infiltration analysis of differential genes in TCGA-BLCA. (E) Masson staining of UTUC and BC.

Supplementary Figure 3

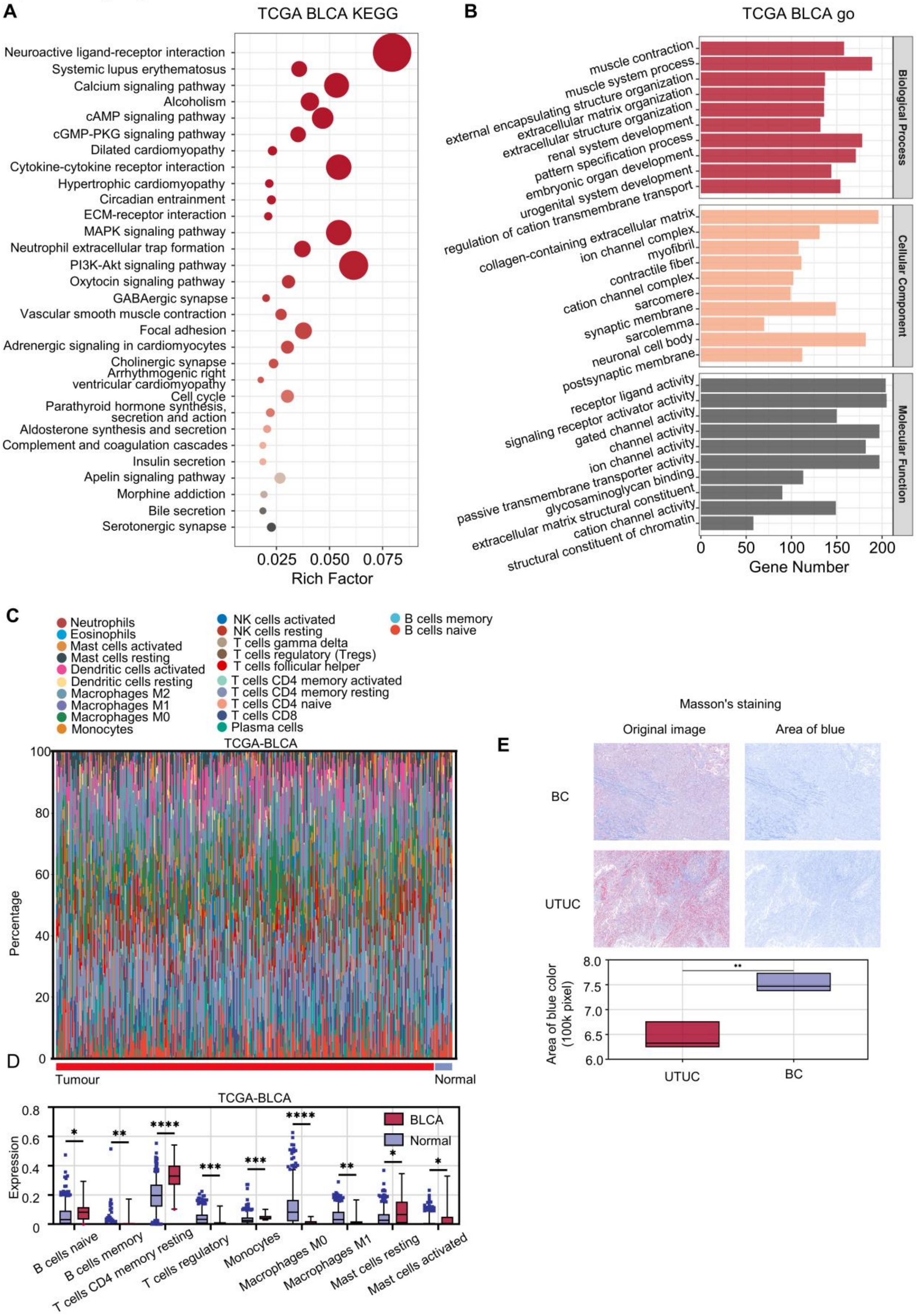

Supplementary Figure 3. Enrichment analysis and immune infiltration of BLCA differential genes.

(A) Heatmap and volcano plot of differential genes in UTUC patient data with a history of Aristolochic acid intake that we collected. (C, D) Heatmap and volcano plot of differential genes in GSE47702. (E, F) Heatmap and volcano plot of differential genes in GSE166912 ($p \leq 0.01$ as **).

Supplementary Figure 4

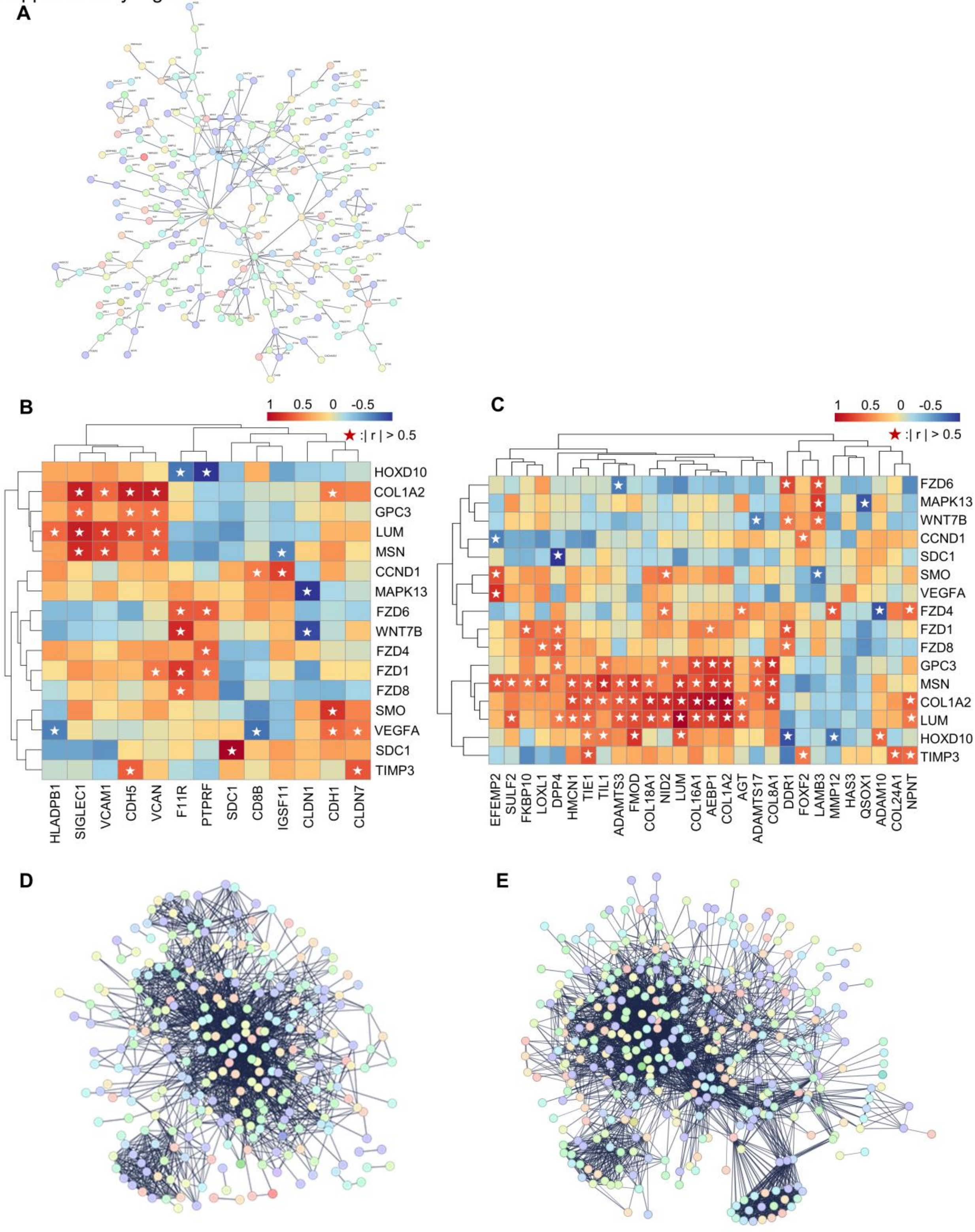

Supplementary Figure 4. Supplementary figures for correlation analysis.

(A) PPI network of UTUC-specific genes. (B, C) Correlation analysis of UTUC-specific genes in the proteoglycan pathway and cell adhesion pathway with ECM pathway in GSE47702. (D) Complete KEGG proteoglycan pathway and cell adhesion pathway PPI network. (E) Complete proteoglycan pathway and ECM pathway PPI network.

Supplementary Figure 5

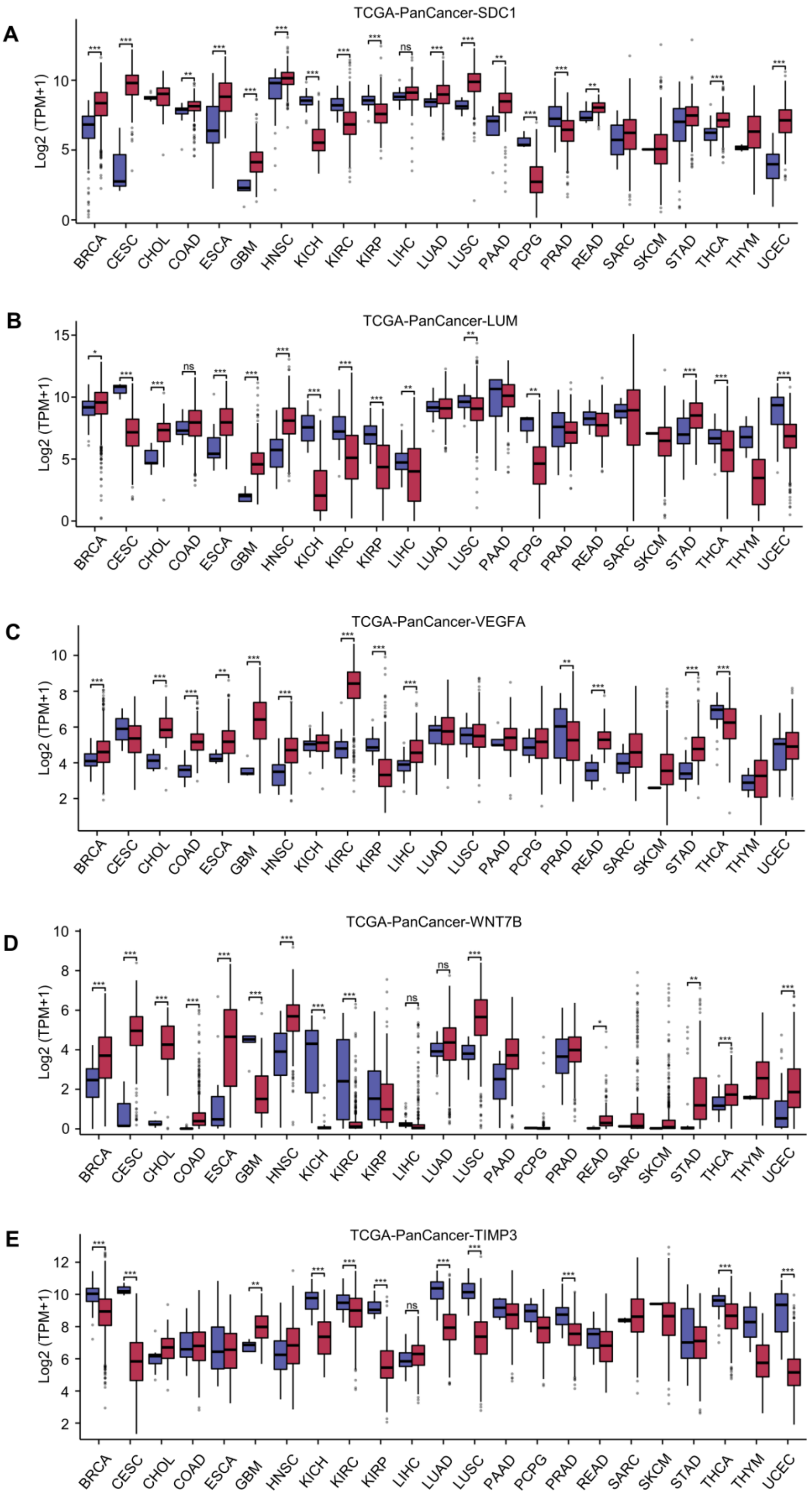

Supplementary Figure 5. Pan-cancer analysis of UTUC-specific differential genes. (A) Pan-cancer analysis of SDC1. (B) Pan-cancer analysis of LUM. (C) Pan-cancer analysis of VEGFA. (D) Pan-cancer analysis of WNT7B. (E) Pan-cancer analysis of TIMP3 ($p > 0.05$ as ns, $p \leq 0.05$ as *, $p \leq 0.01$ as **, $p \leq 0.001$ as ***, $p \leq 0.0001$ as ****).

Supplementary Figure 6

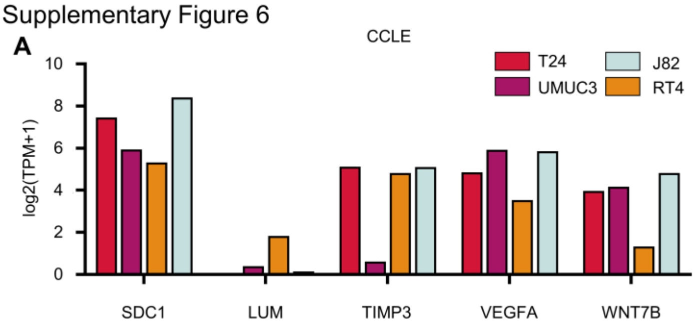

Supplementary Figure 6. Screening of urothelial carcinoma cell lines.
(A) Cell line screening from the CCL database.
